# Partial splenectomy after preoperative embolization in a patient with metastatic melanoma – A case report

**DOI:** 10.1016/j.ijscr.2022.106837

**Published:** 2022-02-17

**Authors:** Tobias Hauge, Eric Dorenberg, Mariusz Goscinski

**Affiliations:** a Dept of Gastrointestinal Surgery, Oslo University Hospital, Radiumhospitalet, PO 4953 Nydalen, 0424 Oslo, Norway; b Division of Radiology and Nuclear Medicine, Oslo University Hospital, Rikshospitalet, PO 4950 Nydalen, 0424 Oslo, Norway

**Keywords:** PS, partial splenectomy, DSA, digital subtraction angiography, PVA, polyvinyl alcohol, RFA, radiofrequency ablation, Partial splenectomy, Superselective embolization, Melanoma, Splenic metastasis, Case report

## Abstract

**Introduction and importance:**

There is lack of evidence regarding the best treatment option for metastatic melanoma. In patients with a single splenic metastasis, preoperative superselective embolization followed by partial splenectomy (PS) could be a feasible treatment strategy to preserve splenic function and hopefully reduce the risk of postoperative bleeding. To our knowledge, this two-step procedure has yet not been published in patients with splenic metastasis.

**Case presentation:**

We present the case of a 73-year-old man with stage IV melanoma consisting of a single splenic metastasis located at the lower pole. Four days prior to surgery, the patient underwent percutaneous superselective embolization of the segmental arteries going to the lower splenic pole. Subsequent, PS was performed using an upper midline laparotomy were a clearly visible tumor was found at the devascularized lower third of the spleen. The splenic parenchyma was divided using an energy device and hemostasis was secured with diathermia and a hemostatic patch. The patient had an uncomplicated recovery and was discharged home on postoperative day 8. Histology revealed an 8 mm, partly necrotic metastasis from a melanoma. There were no signs of recurrency at his last control four months postoperative.

**Clinical discussion:**

There are no guidelines on how splenic metastasis from melanoma are to be removed, nor any literature on postoperative splenic function or survival after PS.

**Conclusion:**

Superselective embolization followed by PS for metastatic melanoma could be a feasible treatment approach in highly selective patients where there is a strong desire to preserve splenic function.

## Highlights

• Total splenectomy is associated with an increased risk of infections, thromboembolism and certain malignancies
• Partial splenectomy (PS) may be considered to preserve splenic function in cases where a splenic lesion is to be removed
• Preoperative superselective arterial embolization can be used to devascularize the relevant splenic segment prior to PS
• To our knowledge, superselective splenic embolization followed by PS has not been published in patients with splenic metastasis

## 1 Introduction

The main functions of the adult spleen are removal of damaged red blood cells, pathogens and production of immune cells to fight antigens. Splenectomy is associated with an increased risk of infections, thromboembolism, certain solid tumors and hematological malignancies [1]. Studies have shown that by preserving at least 25–30% of the splenic parenchyma, adequate immunologic function will be preserved [2], [3]. Thus, spleen-preserving surgery seems favorable. Prior to partial splenectomy (PS) the relevant segment must be devascularized. Typically, this has been done with one out of two techniques: 1) division of the splenic artery and vein (blood supply to the remaining spleen will be provided by the gastric vasa brevia or left gastroepiploic artery) or 2) hilus dissection with ligation of the splenic vessel branches feeding the relevant segment [4]. Both techniques have their disadvantages – either due to inadequate collateral circulation leading to complete splenic infarction or bleeding associated with hilar dissection/incomplete segment-devascularization [4]. Our intention with preoperative superselective embolization was to achieve a safer and more precise devascularization of the segment to be removed. Very little is known about this two-step technique with superselective embolization followed by PS, especially in cases with splenic metastases [4].

In this case report we present the history of a patient with metastatic melanoma consisting of a single splenic metastasis treated with preoperative superselective embolization prior to PS.

This case report has been reported in line with the SCARE Criteria [5].

## 2 Presentation of case

A 73-year-old, previously healthy non-smoking man, using no medications and with no allergies, had a cutaneous melanoma removed in 2013. Five years after the initial removal, a new cutaneous metastasis was diagnosed and removed. Further investigations revealed a total of four metastases, one in each lung, one at the lower- and one at the upper part of the spleen. The patient wanted treatment with Argon-Helium ablation. Since this is a non-standardized treatment approach for melanoma in Norway, he was treated abroad with ablation of both the lung and spleen metastases in 2018. Thereafter he was given 480 mg Nivolumab (PD1-inhibtor) every 4 weeks for a total of two years. He responded well, and all but one of the metastases stopped growing. The lesion at the lower splenic pole continued to grow and was therefore treated with stereotactic radiotherapy consisting of 15 Gy × 3 in January 2020. Upon renewed evaluation in the spring of 2021, no remaining metastases besides the lesion at the lower splenic pole were discovered and he was referred to surgery.

### 2.1 Investigations

**Fig. 1:**
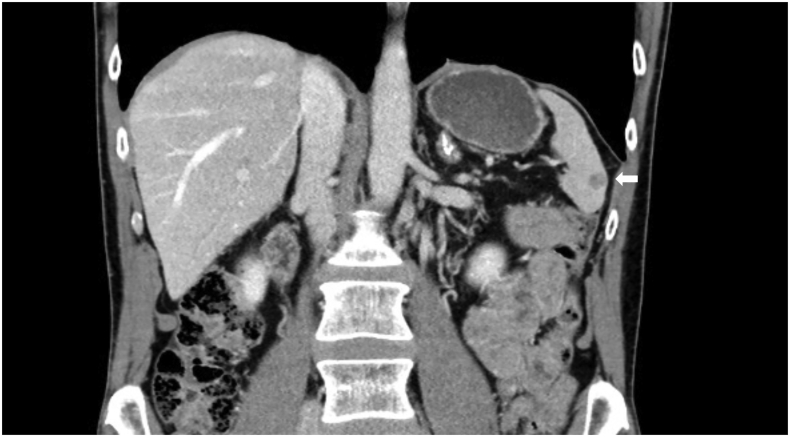
CT-image showing the splenic metastasis to be removed (white arrow).

At the time the patient was diagnosed with metastatic melanoma (in 2018), CT and PET-CT revealed a 17 × 13 mm metastasis at the upper splenic pole and a 6 × 5 mm metastasis at the lower splenic pole. He responded well to Argon-Helium ablation and Nivolumab (PD1-inhibitor) and in November 2019 the lesion at the lower splenic pole was the only growing lesion now measuring 17 mm. A renewed PET-CT in April 2021 revealed no remaining metastases besides the lesion at the lower splenic pole (Fig. 1).

### 2.2 Treatment

Due to the risk of peroperative conversion to total splenectomy, the patient was preemptively vaccinated with a conjugated pneumococcal vaccine (Prevenar), a meningococcal group B vaccine (Bexsero) and a meningococcal group A, C, W, 135 and Y vaccine (Nimenrix) three weeks prior to surgery.

**Fig. 2:**
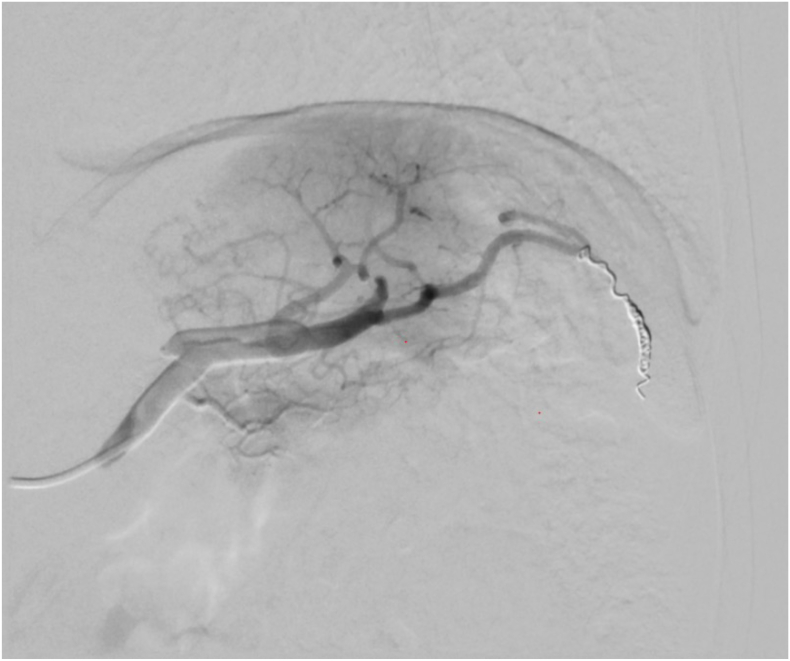
Digital subtraction angiography (DSA) image after PVA particle embolization of the lower pole and coil embolization of the collateral artery.

Four days prior to surgery he underwent superselective arterial embolization of the segmental arteries to the lower splenic pole using polyvinyl alcohol (PVA) particles sized 150–250 μm. A collateral artery communicating with the middle colic artery was embolized with microcoils. After an uncomplicated procedure, angiography confirmed ceased circulation to the lower third of the spleen (Fig. 2). At the time of admission, he was complaining about left upper quadrant pain, needing opioids to ease the pain. His blood tests revealed a hemoglobin of 15.6 g/dL, a leukocyte count of 9.2 × 10^9^/L and a CRP of 114 mg/L. The abdominal pain and the elevated CRP were attributed to segmental splenic ischemia.

**Fig. 3:**
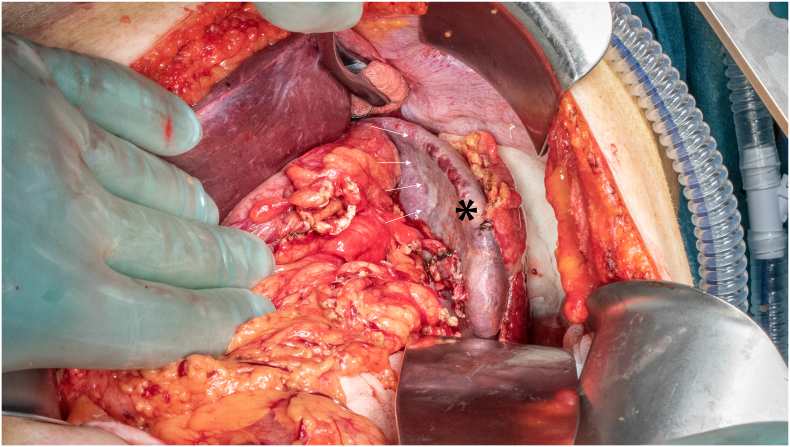
The devascularized lower third of the spleen (*). Ischemic line (white arrows).

**Fig. 4:**
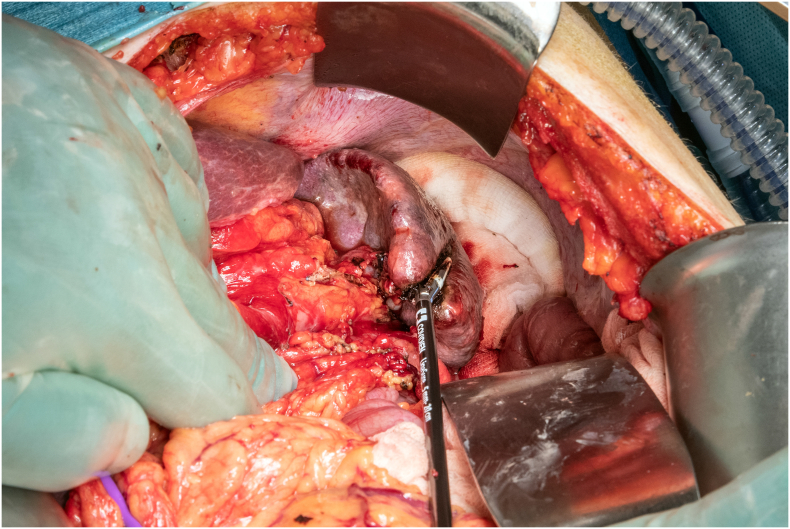
Division of the splenic parenchyma using LigaSure™.

**Fig. 5:**
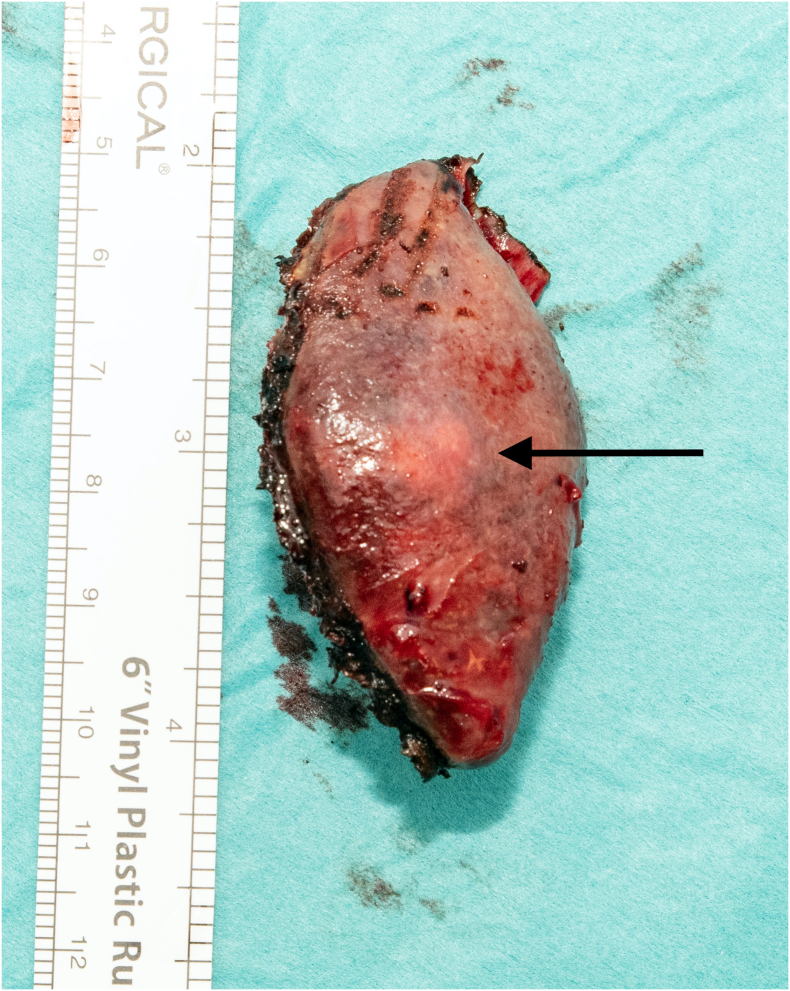
The final specimen, measuring 5.4 × 2.5 cm. The tumor is located at the center (black arrow).

PS was conducted under general anesthesia using an upper midline laparotomy. Author 3 was the surgeon, author 1 the assistent, while the preoperative embolization was conducted by author 2. Confirming the preoperative CT scan, a 1 cm subcapsular tumor was found about 2 cm cranial to the lower splenic pole. The lower third of the spleen was clearly demarcated and ischemic after the embolization, leaving a vital 2/3 of the spleen intact (Fig. 3). In order to diminish the risk of postoperative splenic torsion, only the lower pole was medialized by cutting the peritoneal reflection. The line of resection was marked with good margins to the tumor using diathermia, leaving 1–2 cm of ischemic tissue left before the splenic parenchyma was divided using LigaSure™ Maryland (Medtronic) in a two-step procedure – initially with the jaws half-closed, then fully closed (Fig. 4). We experienced no bleeding. Finally, the raw transection surface was coagulated using diathermia (100 W, “spray” function), then covered with a hemostatic patch (Tachosil™, Takeda AS). The removed splenic tissue (Fig. 5) was sent for frozen section biopsy which revealed necrotic splenic tissue and atypical cells. A 18Fr Blakes drain was placed along the remaining spleen. The total operation time was 130 min, including some waiting on the pathology report.

### 2.3 Outcome and follow-up

The patient experienced a slightly slow recovery, primarily due to low nutritional intake. In order to prevent deep vein thrombosis, he had intermittent pneumatic compression devices around his legs the first postoperative days. Due to fear of bleeding, low-molecular heparin (Deltaparin) was first started on postoperative day 3. There were no complications and he was discharged home on postoperative day 8. The final histology report revealed an 8 mm, partly necrotic metastasis from a melanoma.

At his last check up, 4 months after PS, a PET-CT scan revealed no signs of local splenic recurrency nor other signs of residual melanoma activity. The patient was satisfied with the final result.

## 3 Discussion

The 2019 European consensus-based interdisciplinary guideline on melanoma recommends that surgery should be considered in cases of oligometastatic disease where complete resection is feasible [6]. The guidelines do not specify how the metastases are to be removed. Wood et al. found in a retrospective study of 838 patients with metastatic melanoma that 8% had splenic metastases. In this group, the majority underwent non-operative treatment and had a median survival of 14 months [7]. Only a small, highly selective group, had surgery with a median survival of 28 months. De Wilt et al. reported similar long-term survival of 23 months in seven patients who underwent total splenectomy for a solitary lesion [8].

In a systematic review from 2019 in which 2130 patients were treated with PS, only 88 cases were found to have a non-hematological neoplastic etiology [4]. 81% of all cases were operated with laparotomy, with a mean operation time of 126.5 min and a mean blood loss off 162 mL (0–1200 mL). Peroperatively, a total of 17 complications were registered, including 14 cases of conversion to total splenectomy primarily due to ischemic splenic remnants. Postoperatively, 237 complications (11.1%) were registered, including 12 re-operations with removal of the remaining spleen, primarily due to bleeding, and three deaths because of sepsis (two patients) and colonic perforation (one patient).

For splenic metastasis, we have not found any studies comparing the outcome after partial and total splenectomy, nor the degree of remaining splenic function after PS. In a review article comparing PS and total splenectomy in children operated for congenital hemolytic anemia (sickle cell disease, thalassemia, hereditary spherocytosis), splenic function was preserved short time after PS [9]. Further, a low mortality and bleeding rate and a moderate rate of infections were found postoperative in both PS and total splenectomy. High-quality studies, including long time follow-up and comparison between laparotomy and laparoscopy are missing.

The decision to perform PS in our patient, thus leaving the upper splenic part, which previously contained a now successful ablated metastasis was thoroughly discussed at a local MDT meeting and with the patient. The main reason for choosing this strategy was patient which. The patient was well informed about the probably increased risk of splenic cancer recurrence, but despite that wanted a spleen-preserving procedure in order to utilize a non-documented and only theoretically increased effect on adjuvant immunotherapy.

Both PS and total splenectomy can be done by laparoscopy and laparotomy. However, the procedures have not been compared in a RCT. Laparoscopic splenectomy is associated with longer operation time, but lower surgical mortality (0.2% vs 1%), shorter hospital stay and less perioperative blood loss, thus being the standard approach in many departments [10], [11]. PS is on the other hand in >80% of the cases done by laparotomy, but since 1995 and 2010 laparoscopic and robotic PS, respectively, are being conducted [4].

The patient was initially referred to our departments laparoscopic center that i.a. does laparoscopic splenectomy. Though, due to the nature of the surgery (PS) they rejected him and he was sent to us for a second opinion. Unfortunately, we don't have experience with laparoscopic splenectomy, thus the open approach was decided upon.

The main reason for choosing the LigaSure™ Maryland (Medtronic) as an energy device, was personal preferences in particular with previous liver resections.

We only found one article that describes the outcome after PS for splenic metastasis from melanoma [12]. In this case report, the parenchyma was successfully divided using radiofrequency ablation (RF) without accessing the splenic vessels. We did not find any reports on patients treated with preoperative superselective embolization prior to PS for a malignant disease, but two cases of embolization prior to PS for benign conditions [13], [14].

Partial splenectomy might be feasible in order to reduce the adverse effects of total splenectomy, including infections, thromboembolisms and certain malignancies. In case of malignant splenic lesion, the risk of a later local recurrence following PS is unknown. Whether PS in combination with adjuvant immunotherapy will increase survival as compared to those undergoing total splenectomy followed by immunotherapy and whether preoperative superselective embolization reduces the risk of postoperative bleeding/conversion to total splenectomy must be addressed in randomized control studies.

## Sources of funding

The authors received no financial support for the research, authorship, and/or publication of this article.

## Ethical approval

This case report was conducted according to the declaration of Helsinki.

## Consent

A written informed consent was obtained from the patient for publication of this case report and accompanying images. A copy of the written consent is available for review by the Editor-in-Chief of this journal on request.

## Authors' contribution

TH was the main author of this manuscript and took part in the operation. ED performed the preoperative embolization and participated in writing this manuscript. MG operated the patient and participated in writing this manuscript.

## Research registration

Not applicable.

## Guarantor

Tobias Hauge.

## Provenance and peer review

Not commissioned, externally peer-reviewed.

## Declaration of competing interest

The authors have no conflict of interest to declare.
